# A Self‐Oscillated Organic Synapse for In‐Memory Two‐Factor Authentication

**DOI:** 10.1002/advs.202401080

**Published:** 2024-03-23

**Authors:** Shuzhi Liu, Xiaolong Zhong, Yuxuan Li, Bingjie Guo, Zhilong He, Zhixin Wu, Sixian Liu, Yanbo Guo, Xiaoling Shi, Weilin Chen, Hongxiao Duan, Jianmin Zeng, Gang Liu

**Affiliations:** ^1^ Department of Micro/Nano Electronics School of Electronic Information and Electrical Engineering Shanghai Jiao Tong University Shanghai 200240 China; ^2^ School of Chemistry and Chemical Engineering Shanghai Jiao Tong University Shanghai 200240 China

**Keywords:** in‐memory asymmetric encryption, in‐memory two‐factor authentication, organic synapse, self‐oscillated characteristic

## Abstract

Entering the era of AI 2.0, bio‐inspired target recognition facilitates life. However, target recognition may suffer from some risks when the target is hijacked. Therefore, it is significantly important to provide an encryption process prior to neuromorphic computing. In this work, enlightened from time‐varied synaptic rule, an in‐memory asymmetric encryption as pre‐authentication is utilized with subsequent convolutional neural network (ConvNet) for target recognition, achieving in‐memory two‐factor authentication (IM‐2FA). The unipolar self‐oscillated synaptic behavior is adopted to function as in‐memory asymmetric encryption, which can greatly decrease the complexity of the peripheral circuit compared to bipolar stimulation. Results show that without passing the encryption process with suitable weights at the correct time, the ConvNet for target recognition will not work properly with an extremely low accuracy lower than 0.86%, thus effectively blocking out the potential risks of involuntary access. When a set of correct weights is evolved at a suitable time, a recognition rate as high as 99.82% can be implemented for target recognition, which verifies the effectiveness of the IM‐2FA strategy.

## Graphical abstract

In‐memory two‐factor authentication (IM‐2FA) is implemented to enhance the security of artificial neural networks. Upon successful completion of the initial authentication, where a set of accurate weights is verified, subsequent target recognition is authorized with an impressive recognition rate of 99.82%. However, in cases where incorrect weights are provided for neuromorphic computing, access is denied.

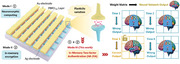

## 1 Introduction

**Figure 1:**
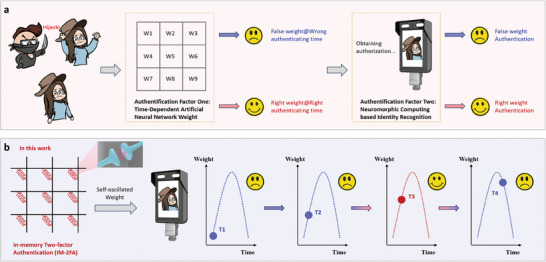
a) Conventional two‐factor authentication (2FA) process facing the hijack situation. b) Schematic illustration of IM‐2FA function utilizing a self‐oscillated synapse with time‐dependent weight variation as the first‐step authentication and subsequent neuromorphic recognition from the neural network as two‐step authentication.

Organic synaptic devices constitute a critical component of neuromorphic electronics.^[^ ^1^, ^2^, ^3^ ^]^ They harness the conductance evolution dynamics of organic molecules to emulate the weight modulation characteristics, along with the neural signal recording, processing, and transmitting functions of biological synapses, showcasing substantial application potential in bio‐inspired computing areas, such as face recognition,^[^ ^4^, ^5^ ^]^ speech analysis,^[^ ^6^, ^7^ ^]^ and fingerprint authentication.^[^ ^8^, ^9^ ^]^ However, when the target has been hijacked, involuntary access will lead to severe consequences, including threats to life and property loss. To resolve this issue, implementing an encryption process prior to object recognition becomes imperative for judging whether the target is in a normal situation or not with a triggered alarm (**Figure** **1**a). It requires a two‐step approach, involving the initial encryption phase followed by subsequent neuromorphic computing process to establish an in‐memory two‐factor authentication (IM‐2FA) strategy, thereby enhancing security in the face of involuntary access.

The state‐of‐the‐art synaptic device has made significant progress in the encryption realm. By virtue of the inherent conductance variation characteristic, the synaptic array can serve as natural physical unclonable functions (PUFs) for hardware encryption. The AND, OR, and XOR logic algorithms are normally adopted to process the data between the input and PUFs.^[^ ^10^, ^11^, ^12^ ^]^ The correct output can be only generated when the input conforms to the rule of PUFs’ key. On the other hand, based on the device's inner high/low conductance state as logic “1”/“0”, the sequence of readout conductance states indicates some implications in the ASCII code.^[^ ^13^, ^14^, ^15^ ^]^ After modulation of the conductance states, the data storage in devices will be modified with encryption to fulfill the in‐memory encryption application. Note that these cutting‐edge hardware encryption methods are typically implemented as separate structures from the neuromorphic computing module. Compared to a single module, these two units will significantly augment the system's complexity and elevate energy consumption during the data transfer process^[^ ^16^ ^]^ (Figure 1a).

The weight within in situ storage and evolution in synaptic devices is a function of time. Leveraging this characteristic, the temporal variation of weights can be considered as the encryption process, with subsequent adaptation to correct weights intended for neuromorphic computing. Together, these processes form the basis of the IM‐2FA within a synaptic device. Herein, we employ a biocompatible polymer, poly(butylene furandicarboxylate)_90_‐b‐(ε‐caprolactone)_10_ (PBFCL_10_), as the dielectric layer to fabricate the Au/PBFCL_10_/Ag  synaptic device. A self‐protective depression (SPD) phenomenon subsequent to long‐term potentiation (LTP) can be observed with a remarkable linear conductance evolution during self‐oscillated cycles. Compared with bipolar pulse modulation, self‐oscillated unipolar bias can significantly decrease the complexity of the peripheral circuit.^[^ ^17^ ^]^ Additionally, spike‐timing‐dependent plasticity (SRDP) can be modulated by different pulse intervals (20, 30, 40, 80, 120, and 200 µs). The mechanism of the SPD process can be ascribed to the thermal energy‐induced evolution of Ag filament. An in‐memory asymmetric encryption application has successfully achieved between the plaintext “Hello” and the ciphertext “Chemy” based on the self‐oscillated property with two different public and private keys for encryption and decryption processes, respectively. Based on encryption, we construct a ConvNet to attain a proof‐of‐concept IM‐2FA. The results demonstrate that without passing the encryption at the appropriate time, the ConvNet achieves a low recognition rate ranging from 0.37% to 0.86%. However, at the correct time with suitable weights, a high recognition rate of 99.03%–99.82% is attained for digital numbers from 0 to 9. Therefore, the IM‐2FA application can be achieved.

## 2 Results and Discussion

### 2.1 The Design Essence of the In‐Memory Two‐Factor Authentication

Synaptic plasticity, governed by dynamic variations of neurotransmitters, constitutes a vital mechanism within the intricate web of the nervous system.^[^ ^18^ ^]^ It orchestrates the fine‐tuning of synaptic connections between neurons, facilitating in situ data storage and processing.^[^ ^19^, ^20^ ^]^ This phenomenon has garnered extensive attention in the realms of cognitive science, computational neuroscience, and spiking neural networks.^[^ ^21^, ^22^, ^23^ ^]^ Notably, synaptic plasticity manifests in different forms with a function of time, distinguished by the duration of their effects and their operational methodologies. Examples include short‐/long‐term potentiation (S/LTP),^[^ ^24^, ^25^ ^]^ short‐/long‐term depression (S/LTD),^[^ ^26^, ^27^ ^]^ spike‐timing‐dependent plasticity (STDP),^[^ ^28^, ^29^ ^]^ and SRDP.^[^ ^30^, ^31^ ^]^ Therefore, by virtue of time‐dependent dynamic characteristics (From T1 to T4), in‐memory encryption can be designed as a pre‐authentication system for subsequent target recognition as an IM‐2FA accomplishment (Figure 1b). Upon passing the encryption with suitable weight at the correct time (T3), authentication for the utilization of neuromorphic computing is achieved. Otherwise, pre‐authentication fails with a backend alarm (T1, T2, and T4).

### 2.2 The Self‐Oscillated Synaptic Performance of the Au/PBFCL_10_/Ag Device

**Figure 2:**
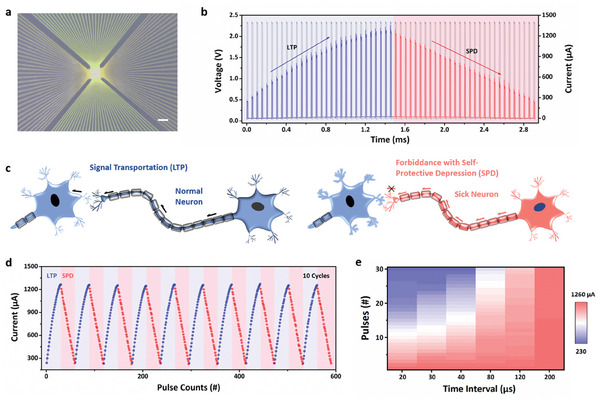
a) The scanning electron microscopy images of the Au/PBFCL_10_/Ag memristive device. Bar scale: 100 µm. b) The LTP and SPD effects of the Au/PBFCL_10_/Ag device with continuous 59 pulses (amplitude: 2.3 V, width: 10 µs, and interval: 40 µs). c) The schematic diagram of the signal transportation in normal neuron and signal forbiddance in sick neuron by post‐synapse, corresponding to LTP and SPD effects in Figure 2b, respectively. d) The self‐oscillated behavior of the Au/PBFCL_10_/Ag synaptic device. e) The variation amplitude of the current under the pulse stimulation with different time interval.

To attain the aforementioned level of performance, we utilized UV lithography technology to fabricate Au/PBFCL_10_/Ag device with a 2 µm line width (**Figure** **2**a). The PBFCL_10_ has successfully synthesized with the copolymerization method and detailedly characterized with the gel permeation chromatography (GPC), nuclear magnetic resonance (NMR), phase separation‐based measurement, and differential scanning calorimetry (DSC) summarized in Figures S1–S5 and Table S1 (Supporting Information). The polymer has been evidenced to have biocompatible behavior with no cytotoxicity, indicating its promising potential to develop brain‐computer interface (BCI) (Figures S6–S7, Supporting Information). With the spin‐coating on the Si/SiO_2_ substrate, the PBFCL_10_ shows a smooth surface with a 0.39 nm root‐mean‐square (RMS) roughness (Figure S8, Supporting Information). The zoom‐in mode and the cross‐sectional structure of the device are displayed in Figure S9 (Supporting Information). All the electrical measurements have been applied on the bottom Ag electrode and set the top Au electrode grounded. Regarding the functional level, the process of the active Ag electrode undergoing oxidation reaction to inject silver ions toward the counter Au electrode resembles the release of neurotransmitters from the presynaptic to the postsynaptic membrane, endowing the device with synapse‐like performance. Initially, the Au/PBFCL_10_/Ag device shows a low conductive characteristic with 240.6 µA. Upon applying a pulse on the PBFCL_10_‐based device (amplitude of 2.3 V, width of 10 µs, and interval of 40 µs), the device current increases from 240.6 to 276.01 µA, indicating short‐term potentiation (STP). After that, applying the other 28 pulses of stimulation, the device will transfer STP into a LTP, with the current continuously increasing from 276.01 to 1251.4 µA. However, abnormally, without changing the pulse polarity after 29 pulses, the other 29 pulses will decrease the device current from 1251.4 to 232.2 µA with a SPD, as shown in Figure 2b, which is similar to the current decrease process of long‐term depression (LTD) normally with a reversed pulse polarity. This unique feature, tuning the current value by the same pulse polarity with LTP and SPD, has an analog to the self‐protective mechanism in the real biology neural system to interdict the morbid signal. As shown in the left plane of Figure 2c, for a normal neuron, the electrical signal transfer from the pre‐neuron to post‐neuron will cause a synaptic weight increase (conductance in electronic device) implying the connection between them rising, like the result of the first 30 pulses stimulation in Figure 2b. As for a sick neuron, substantial pulse signals have transferred to the post‐neuron, during which the post‐synapse will judge the pre‐neuron has sicked with an irrational stimulation sending reversed pulses to counteract that effect (right plane of Figure 2c), like the pulses stimulation from the 31^st^ to 59^th^ in Figure 2b. The device can continuously operate for 10 cycles of the LTP and SPD with a self‐oscillated characteristic, showcasing an excellent conductance modulation behavior (Figure 2d). The conductance uniformity of 99.78% during 10 cycles can be calculated by the equation as shown below:

(1) $$Uniformity\;\left(\%\right)\;=\;1\;-\;\left(\frac{\sum_{i\;=\;1}^{59}{\left(\frac{\delta }{\mu }\right)}_{i}}{59}\;\times \;100\%\right)$$

where *i* is the sequence number of pulses, δ is the standard deviation and μ are the average values of the current values in ten cycles. Such high cycle‐to‐cycle modulation uniformity during the self‐oscillated process can meet the basic requirement of practical applicability. During the LTP process, the current variation has fitted with the exponential function in Figure S10 (Supporting Information) and a linear fitting during the SPD process can be observed with the equation (y = 1294.69 × –35.15). By regulating the pulse intervals with 20, 30, 80, 120, and 200 µs after LTP, the SPD can be carefully modulated toward the amplitude of current decrease (Figures S11 and S12, Supporting Information). Therefore, SRDP during the SPD process can be modulated with the double exponential function, fitted as below Equation (2) (Figure S13, Supporting Information):

(2) $$\Delta \omega =C_{1}\exp\left(-t/\tau_{1}\right)+C_{2}\exp\left(-t/\tau_{2}\right)$$

where Δ*ω* is the change ratio of synapse weight (device conductance), C_1_ and C_2_ are the constants of 0.81 and 0.65, *t* is the time interval between neighboring pulses, *τ*_1_ and *τ*_2_ are time constants of the SRDP curves, derived to 20.22 and 114.11 µs, respectively. The results display that with pulse interval increases as a low‐frequency pulse, the decreased amplitude will slacken, while with pulse interval decreases as a high‐frequency pulse, the decreased amplitude will become pronounced (Figure 2e; Figure S14, Supporting Information), both of which have conformed to the self‐protective mechanism in the real biological system.^[^ ^32^ ^]^

### 2.3 Mechanism of PBFCL_10_ Synaptic Device

**Figure 3:**
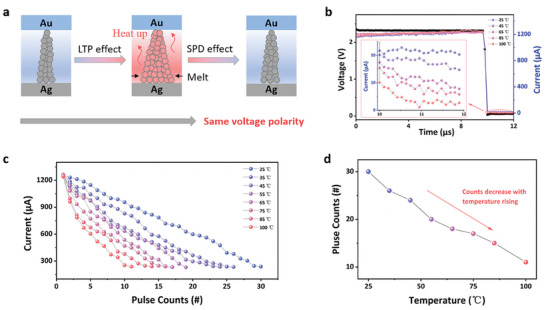
a) Schematic illustration of the physical mechanism based on the Au/PBFCL_10_/Ag device during the LTP and SPD processes. The current variation from 25 to 100 °C under b) one pulse stimulation (amplitude: 2.3 V, width: 10 µs, and interval: 40 µs) and c) continuous pulses stimulation to the low conductance state. d) The demanded pulse counts at different temperature during SPD process.

The electrochemical metallization memory (ECM), employing active electrodes like Ag and Cu, has been widely utilized for emulating synaptic behavior.^[^ ^33^, ^34^, ^35^, ^36^, ^37^ ^]^ It leverages processes such as oxidization‐induced ion injection, directed migration toward negative bias, and reduction‐induced conductive filament formation to modulate electronic transportation behavior. Traditionally, the generation and degeneration of nanoscale conductive filaments necessitate the application of opposite polarity voltages to regulate cation migration. However, only a few studies have reported that filament degeneration can be achieved by utilizing the same polarity voltage as the degeneration process. In this section, we will elucidate the mechanism based on Au/PBFCL_10_/Ag during the working processes of LTP and SPD. Initially, in accordance with quantized conduction theory, the device's conductance of 1.3 G_0_ (equivalent to 240.6 µA) reaches the size of an atomic point contact and is quantized in half‐integer units of G_0_ = 2*e* ^2^/*h* = 77.5 µS (where *e* represents the elemental charge of electrons, and *h* is the Planck's constant) (left plane of **Figure** **3**a). With successive pulse stimulation, cations migrate toward the negative bias and undergo reduction, resulting in the formation of a thick conductive filament with a current of 1251.4 µA or 7.0 G_0_ (middle plane of Figure 3a). However, as the conductive electron ability increases, the current flowing through the device becomes more pronounced, accompanied by the Joule heat effect (Q = I^2^Rt). Consequently, a thermally driven conductive filament gradually melts or undergoes spontaneous dissolution, leading to a noticeable decrease in conductance and the manifestation of the SPD effect (right plane of Figure 3a). To substantiate this physical evolution process, we designed an in‐operando temperature‐controlled electrical measurement. Following a transient current response of 1222.2 µA observed at room temperature with a single 2.3 V pulse stimulation, a current of 20.34 µA can be sustained at room temperature for 2 µs with a read voltage of 0.05 V (Figure 3b). Upon increasing the operating temperature to 45 °C, a significant decrease in current from 20.21 to 14.78 µA is observed, indicating that thermal energy hampers the generation of the silver conductive filament. Subsequently, as the temperature is raised to 65, 85, and 100 °C, thereby providing more pronounced thermal energy, the current further decreases to 7.83, 5.81, and 2.70 µA, respectively. These results indicate that the silver conductive filament can readily dissolve or exhibit spontaneous thermal motion with increasing temperature. Hence, under the condition of continuous pulse stimulation, the silver atoms at room temperature experience comparatively small‐scale thermal motion, resulting in a gradual decrease in current from 1257.57 to 237.58 µA over 29 continuous pulses (Figure 3c). Upon increasing the operating temperature to 35 °C, the thermal energy from the environment induces more significant thermal motion of the silver atoms, leading to a relatively rapid decrease in current from 1263.45 to 232.79 µA over 26 pulses. Further increasing the temperature to 45, 55, 65, 75, 85, and 100 °C, an obvious amplification of the current decrease is observed with increasing temperature, requiring fewer pulse stimulations to return to the low conductive state: 24, 20, 18, 17, 15, and 11 counts, respectively. For ease of visualization, the relationship between operating temperature and pulse counts is depicted in Figure 3d. In summary, the mechanism underlying the SPD process can be attributed to the thermal energy‐controlled evolution of the silver conductive nanofilament.

### 2.4 The Encryption Achievement

**Figure 4:**
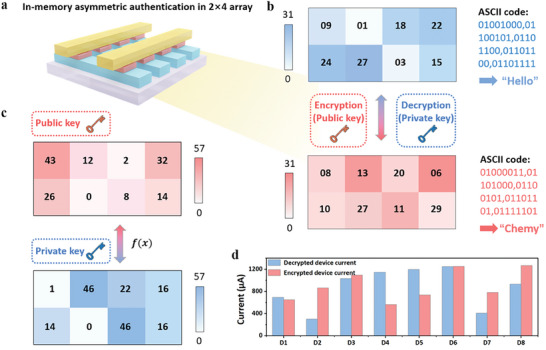
a) The schematic diagram of the 2 × 4 Au/PBFCL_10_/Ag memristive array for in‐memory asymmetric encryption. b) The encryption and decryption operations in the 2 × 4 Au/PBFCL_10_/Ag memristive array. The cleartext of “Hello” and the ciphertext of “Chemy” encoding with the ASCII code maps with the conductance sequence in the 2 × 4 Au/PBFCL_10_/Ag array. “Hello” represents the “09”, “01”, “18”, “22”, “24”, “27”, “03”, and “15” conductance sequence, and the “Chemy” implies the “08”, “13”, “20”, “06”, “10”, “27”, “11”, and “29” conductance sequence. c) The public and private keys during the asymmetric encryption and decryption processes. *f*(*x*) denotes a function of pulse counts according to the Equation (3). d) The histogram of current value in the eight devices at the process of original data storage and encrypted data.

Stating a comprehensive exposition on the fluctuations of self‐oscillated synapse behavior within their internal physical mechanism, in‐memory asymmetrical encryption has been accomplished utilizing the self‐oscillated synaptic plasticity, ingeniously arranged in a 2 × 4 memristive array (**Figure** **4**a). Each device possesses a range of 30 quantized conductance states spanning from 1.3 to 7.0 G_0_, effectively enabling quasi‐5‐bit storage capacity with two vacant binary numerals. To accommodate the storage requirements of the plaintext, namely “Hello,” a meticulous encoding process is employed to transform each letter (“H,” “e,” “l,” “l,” and “o”) into their corresponding 8‐bit binary representations: “01001000,” “01100101,” “01101100,” “01101100,” and “01101111,” respectively. These sequential digit numbers, totaling 40, can be seamlessly stored within the eight devices, utilizing a 5‐bit binary transfer scheme, characterized by their conductance sequences denoted as “09,” “01,” “18,” “22,” “24,” “27,” “03,” and “15,” respectively (top plane of Figure 4b). In order to safeguard the integrity of the data, a public key is generated through pulse stimulation, adopting a sequential pattern: 43, 12, 2, 32, 26, 0, 8, and 14 counts (top plane of Figure 4c). Subsequently, their current is modulated, transitioning from 693.53 to 652.53 µA, 300.53 to 864.46 µA, 1032.03 to 1094.26 µA, 1145.25 to 562.25 µA, 1195.29 to 736.78 µA, 1248.69 to 1251.56 µA, 408.24 to 780.13 µA, and 933.90 to 1266.26 µA, respectively (Figure 4d). Consequently, the encrypted data undergoes a transformation, adopting a conductance sequence comprising “08,” “13,” “20,” “06,” “10,” “27,” “11,” and “29”, which corresponds to the converted ASCII code of “01000011,” “01101000,” “01100101,” “01101101,” and “01111101,” thereby conveying the intended meaning of “Chemy” (bottom plane of Figure 4b). By employing the private key, aligned with the appropriate pulse stimulation pattern of 1, 46, 22, 16, 14, 0, 46, and 16 counts, in accordance with the self‐oscillated synapse performance of the device, as demonstrated in Figure 2d, the decryption process can be effectively executed, facilitating access to the plaintext of “Hello” (bottom plane of Figure 4c). The needed switching pulse counts during the encryption and decryption processes conform to Equation (3) as shown below:

(3) $$f\;\left(Pulse\;Counts\right)=\left\{\begin{array}{c} 0,\;C1=C2\\ \left(29-C1\right)+\left(29-C2\right),\;C1>C2\\ C2-C1,\;C1<C2 \end{array}\right.\;$$

where C1 and C2, ranging from 0 to 29, represent the counts of current and modulated conductance states, respectively. Calculated from Equation (3), the largest pulse counts are modulated in the conductance sequence from 1 to 0, requiring 57 pulse stimulations in total, computed as (29‐1) + (29‐0). In comparison to the symmetrical operation, which relies on the same key to encrypt and decrypt the data, the asymmetrical nature of this strategy, leveraging public and private keys derived from the device's self‐oscillated synapse behavior, significantly enhances the efficacy of in‐memory encryption. Consequently, even if the public key were to be compromised by hackers, a reversal of the operation and access to the plaintext remains unattainable without the utilization of a private key. It is worth noting that, 30 conductance states in the Au/PBFCL_10_/Ag memristive device cannot be impeded by the function of 5‐bit in‐memory encryption in this work but lost a partial of binary information.

### 2.5 Proof‐of‐Concept IM‐2FA

Based on the encryption process, a ConvNet can operate a target recognition task to accomplish IM‐2FA. Figure S15 (Supporting Information) provides an overview of the ConvNet structure used in the dynamic authentication process for recognizing the digital number “3”. The ConvNet consists of two sets of convolutional and average pooling layers, followed by a flattened convolutional layer, then a fully‐connected layers, and finally a softmax classifier. These components collaborate to accurately extract features from the input image and classify it. The initial layer represents a grayscale image with dimensions of 28 × 28, serving as the input for subsequent layers. The first convolutional layer applies a convolution operation using a kernel size of 3 × 3, aiding in the detection of local features such as edges and corners by convolving the input image with the filters. The resulting feature maps of size 26 × 26 × 15 then undergo a max pooling with a 2 × 2 filter size and a stride of 2 to introduce translation invariance by reducing the spatial resolution of the feature map while retaining the most significant features. The second convolutional layer employs a 5 × 5 kernel size to detect more complex features by combining the features detected by the previous layers. Similarly, a max pooling operation is performed on the resulting feature maps to introduce translation invariance while retaining the most significant features. the resulting feature maps are then fed into the flattened convolutional layer to obtain 1D vectors, which are subsequently passed to the fully connected layers. Finally, a fully connected layer combines the features detected by the previous layers to make higher‐level decisions about the input image, and a softmax classifier produces class probabilities for each of the 10 possible digits. The network's weights have been trained using the MNIST dataset, which consists of 60 000 training and 10 000 testing images of handwritten numerals ranging from 0 to 9. The ConvNet acquires the ability to accurately recognize and classify handwritten digits after undergoing the training process. Its exceptional classification ability makes it well‐suited for the dynamic authentication process in IM‐2FA, where the ConvNet acts as the second‐step authentication after the first‐step authentication utilizing the self‐oscillation of the synaptic device.

**Figure 5:**
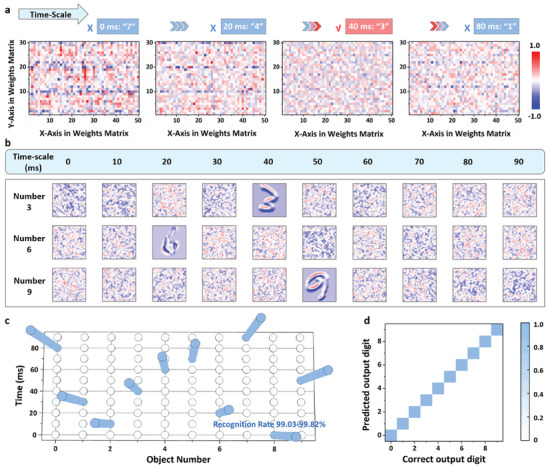
a) The weights evolution of self‐oscillated synaptic device at initial time, 20, 40, and 80 ms with “7”, “4”, “3” and “1” neural network output, respectively. The correct weights at the 40 ms will be authorized to pass the first‐step authentication, while at other time with wrong weights, the request will be denied. b) The feature map evolution (26 × 26) of the hand writing number of 3, 6 and 9 input image with the feature extraction by time. c) The recognition rate of the ConvNet toward the digital numbers 0–9 by time. d) The confusion matrix of the ConvNet for handwriting number recognition from 0 to 9.

The demonstration of mapping the network weights to device conductance is presented in Table S2 (Supporting Information). The network's correct weight matrix is set at 40 ms to authorize the first‐step authentication for recognition of digital number 3. At the beginning, the network is initialized with a set of random weights, resulting in an incorrect network output of “7” with a refusal (**Figure** **5**a‐1). Further, by modulation of each weight in pixel with selected varying rate with SRDP‐enhanced self‐oscillated synapse plasticity, the weight varying in each pixel will be tuned by time. The output at 20 ms also yields an erroneous result of “4” without passing the first‐step authentication of in‐memory asymmetric encryption (Figure 5a‐2). Analogous to a robbery case, an automatic action is triggered, alerting the police to ensure the host's safety. The correct weights are revealed at 40 ms authorizing the first‐step authentication (Figure 5a‐3). Subsequently, the input undergoes a second‐step authentication of target recognition, resulting in the accurate recognition of the digital number “3”. Likewise, if the weights continuously vary to deny access during the first‐step authentication, an incorrect result of “1” is generated at 80 ms (Figure 5a‐4). To provide a visual representation of the network's operation, Figure 5b displays the feature map evolution after the convolutional and pooling processes. With a grayscale image (28 × 28) as the input, the feature map (26 × 26) changes the pulse stimulations recorded from the initial time to 90 ms. These pulse stimulations play a crucial role in authorizing access to the correct feature map extraction with time‐evolutional characteristics. At 40 ms for number “3” recognition, the pulse stimulations reveal a set of correct weights in the convolutional kernel, enabling the extraction of the correct information from the input. However, during other time intervals (0–90 ms except for 40 ms), wrong feature maps will be extracted due to the presence of incorrect weights without passing the first‐step authentication. Changing the preset time, a similar result can be attained at 20 and 50 ms for the numbers “6” and “9” recognition, respectively. Following the ConvNet learning processes for IM‐2FA, a high recognition rate ranging from 99.03% to 99.82% is achieved among digital numbers from 0 to 9 (Figure 5c). This demonstrates the effectiveness of the ConvNet in accurately recognizing the target digits when IM‐2FA is employed. In contrast, without passing the IM‐2FA, a low recognition rate ranging from 0.37% to 0.86% is realized to reject requests from unfamiliar access. To further evaluate ConvNet's objective recognition performance, the accuracy of each class in the confusion matrix for numbers 0–9 is displayed, ranging from 98.84% to 99.59% (Figure 5d). This emphasizes ConvNet's powerful performance in undertaking recognition tasks. Additionally, Figure S16 (Supporting Information) illustrates the evolution of accuracy and the loss value with the variation of the learning epoch, providing insights into the network's learning process and its convergence to optimal performance.

## 3 Conclusion

In summary, to ensure the validity of the target recognition process, we design time‐dependent synaptic plasticity as an encryption process prior to the target recognition with a ConvNet, achieving an in‐memory two‐factor authentication. To this end, we have fabricated a synaptic device with an Au/PBFCL_10_/Ag structure, exhibiting self‐oscillated synapse plasticity characterized by long‐term potentiation along with a noticeable self‐protective depression. By finely adjusting the pulse intervals (20, 30, 40, 80, 120, and 200 µs), we achieve pronounced spike‐rate‐dependent plasticity. The underlying mechanism of the device has been elucidated through in‐operando temperature‐controlled measurements, confirming the mechanism of the self‐oscillated behavior results from the thermal energy‐controlled evolution of Ag conductive filaments. Furthermore, we have successfully implemented asymmetric in‐memory encryption based on self‐oscillated synapse behavior. Finally, IM‐2FA indicates that when passing to the first‐step authentication of in‐memory asymmetric encryption, the ConvNet exhibits a high recognition rate of up to 99.82% for the numbers 0–9. Conversely, without passing through the encryption, the subsequent target recognition will be denied access.

## 4 Experimental Section

### Synthesis of Polymer PBFCL_10_ and Characterization

The organic synapse material PBFCL_10_ has successfully synthesized with the copolymerization method from poly(butylene furandicarboxylate) PBF and poly(*ε*‐caprolactone) PCL in Figure S1 (Supporting Information). Proper amounts of PBF oligomer and PCL diol were added into the reaction container. The targeted copolymer was defined as PBFCL_10_, wherein the subscript 10 meant 10 mol% of CL units in the copolyester. In order to protect the reaction and intermediate products, Sb_2_O_3_ (0.15 wt%) and antioxidant 1010 (0.1 wt%) were put into the reactor. On the other hand, the pressure of the reaction system was kept at 500 Pa for 200 °C, and then it was further decreased to less than 15 Pa during heating to 220 °C to minimize possible thermal degradation and oligomers sublimation. Molecular weights were determined with a Waters 2690 gel permeation chromatography (GPC) using polystyrene standards eluting with tetrahydrofuran. ^1^H nuclear magnetic resonance (NMR) spectra were recorded on a Bruker 400 spectrometer at 400 MHz in deuterated chloroform with tetramethylsilane (TMS) as reference for the chemical shifts. The basic thermal parameters were measured by DSC (Perkin Elmer Diamond DSC) with a heating/cooling rate of 10 °C min^−1^. Atomic force microscopy (AFM) has performed on a Solver P47‐PRO (NT‐MDT Co., Moscow, Russia) microscope to monitor the surface morphology.

### PBFCL_10_‐Based Organic Synapse Device Fabrication and Characterization

The memristive characteristics of PBFCL_10_ were evaluated in a nanometer scale device of Au/PBFCL_10_/Ag in 32 × 32 crossbar arrays. The devices were fabricated through ultraviolet lithography technique and a lift‐off approach. Crossbar electrode strips, with a width of 2 µm and a separation of 1.2 µm between each other, were formed on SiO_2_/Si substrate. The 32 bottom electrode strips were patterned by ultraviolet lithography with a SUSS MA6 Mask Aligner UV photolithography system and E‐beam evaporation of a 35 nm Ag layer on the top of a 5 nm Ti adhesion layer on a Denton Electron Beam Evaporator. After lift‐off, a 20 µL of PBFCL_10_ solution (2 mg mL^−1^) in chloroform was spin‐coated onto the bottom electrodes at 3500 rpm for 60 s. Afterward, the obtained samples were dried at 65 °C in vacuum for 8 h. Finally, the 32 top electrode stripes consisting of 5 nm Ti and 50 nm Au were patterned and deposited using UV photolithography, e‐beam evaporation, and lift‐off similarly. All the electrical measurements of the devices in this work were performed on a Keithley 4200 semiconductor parameter analyzer equipped with a pulse‐measuring unit. The in‐operando temperature measurement has conducted on the Lake Shore Model 336 temperature controller. The optical microscope and scanning electron microscope characterization have utilized the RoHS DM210 camera eyepiece and FEI Sirion 200, respectively.

### ConvNet Stimulation

An IM‐2FA system was developed based on a ConvNet for the task of recognizing handwritten digits in the MNIST database^[^ ^38^ ^]^ (Figure S15, Supporting Information). The dataset comprises 70 000 images of handwritten digits ranging from ‘0′ to ‘9′. Out of these, 60 000 images were allocated for training the network, while the remaining 10 000 images were reserved for testing the network and evaluating its classification accuracy. The original MNIST images were 28 × 28 pixels in size and had intensity values spanning from 0 to 255. The intensity values were rescaled to a range of [0, 1.0] to standardize the data. The initial convolutional layer employed 15 kernels with dimensions of three rows and three columns to filter the input images of size 28 × 28. Subsequently, a max‐pooling layer with a size of 2 × 2 was applied. Another convolutional layer followed with 30 filters and a 5 × 5 kernel as the input. This was succeeded by another max‐pooling layer with identical hyperparameters. Toward the end of the network, two fully connected layers were added, comprising 150 and 10 neurons, respectively, facilitating supervised digit recognition. To mitigate the potential issue of overfitting due to the limited number of samples, a dropout mechanism was integrated with a dropout probability of 0.5 at the first fully connected layers. The Adam optimizer was utilized to minimize the cross‐entropy cost function. The system was implemented using the PyTorch deep learning framework libraries and trained on a GeForce RTX 2080Ti graphics processing unit (GPU).

### Statistical Analysis

In Figure S6 (Supporting Information), the viability assessments of the NIH/3T3 cells after incubated with extract solutions collected from the PBFCl_10_ thin film (sample 1–3) and blank (sample 4) were 97.04% ± 7.38%, 104.61% ± 6.54%, 101.30% ± 7.17%, and 102.21% ± 9.22%, respectively. The sample size was 5.

## Conflict of Interest

The authors declare no conflict of interest.

## Author Contributions

S.L., X.Z., and Y.L. contributed equally to this work. S.L., X.Z., Y.L., J.Z., and G.L. conceived the idea. S.L., Y.L., Z.H., and B.G. synthesized and characterized the polymers. S.L., Y.G., W.C., and H.D. fabricated the memristor devices and conducted the electrical measurements. X.Z., S.L., X.S., and J.Z. performed the time dependent neural network. S.L., X.Z., Y.L., J.Z., and G.L. co‐wrote the paper. All the authors discussed the results and commented on the manuscript.

## Supporting information

Supporting Information

## Data Availability

The data that support the findings of this study are available from the corresponding author upon reasonable request.
