# Supplementary material for: A Self‐Oscillated Organic Synapse for In‐Memory Two‐Factor Authentication
Source: Adv Sci (Weinh). 2024 Mar 23;11(21):2401080. doi: 10.1002/advs.202401080 (PMC11151044; doi:10.1002/advs.202401080)
Supplement: Supplementary file 1 — Supporting Information [file ADVS-11-2401080-s001.pdf]

ADVANCED SCIENCE
Open Access

Supporting Information

for *Adv. Sci.*, DOI 10.1002/advs.202401080

A Self-Oscillated Organic Synapse for In-Memory Two-Factor Authentication

Shuzhi Liu, Xiaolong Zhong, Yuxuan Li, Bingjie Guo, Zhilong He, Zhixin Wu, Sixian Liu, Yanbo Guo, Xiaoling Shi, Weilin Chen, Hongxiao Duan, Jianmin Zeng and Gang Liu**

A Self-Oscillated Organic Synapse for In-Memory Two-Factor Authentication

Shuzhi Liu[1,2†], Xiaolong Zhong[1†], Yuxuan Li[2†], Bingjie Guo[2], Zhilong He[2], Zhixin Wu[1], Sixian Liu[1], Yanbo Guo[1], Xiaoling Shi[1], Weilin Chen[1], Hongxiao Duan[1], Jianmin Zeng[1*], Gang Liu[1,2*]

S. Liu, X. Zhong, Z. Wu, S. Liu, Y. Guo, X. Shi, W. Chen, H. Duan, J. Zeng, G. Liu

Department of Micro/Nano Electronics, School of Electronic Information and Electrical Engineering, Shanghai Jiao Tong University, Shanghai 200240, China.

E-mail：jamy3531@sjtu.edu.cn (Dr. Jianmin Zeng); gang.liu@sjtu.edu.cn (Prof. Gang Liu)

S. Liu, Y. Li, B. Guo, Z. He, G. Liu

School of Chemistry and Chemical Engineering, Shanghai Jiao Tong University, Shanghai 200240, China.

E-mail：gang.liu@sjtu.edu.cn (Prof. Gang Liu)

[†]These authors contribute equally to this work.

Table of Contents

Section 1. The synthesis of the polymer $PBFCL_{10}$..3

Section 2. Characterization of the polymer $PBFCL_{10}$ 5

Section 3. Biocompatibility of the polymer $PBFCL_{10}$..11

Section 4. Morphological property of the $PBFCL_{10}$ film...15

Section 5. Zoom-in mode of the Au/$PBFCL_{10}$/Ag device...16

Section 6. Modulation of self-protective depression plasticity................................17

Section 7. The simulation results for the in-memory two-factor authentication (IM-2FA) achievement....20

Section 1. The synthesis of the polymer $PBFCL_{10}$

Poly(ε-caprolactone) (PCL) diol oligomers (>99%, M_n=2000 g/mol), which are derivatives of natural bioproducts, were provided by Shenzhen Esun Industrial Co., Ltd. (China). Antioxidant 1010, 1,4-Butanediol (BDO), tetrabutyl titanate (TBT), 1,1,1,3,3,3-hexafluoro-2-propanol (HFP), methanol and antimony trioxide (Sb_2O_3) were purchased from Aladdin. 2,5-Furan dicarboxylic acid (FDCA, 99%) was purchased from Chem Target Technologies Co., Ltd. (China). The dimethyl furandicarboxylate (DMFD) was prepared based on FDCA[1]. Phenol and tetrachloroethane were purchased from Aladdin as solvents for viscosity measurements.

Synthesis of poly(butylene furandicarboxylate) PBF (**Figure S1**): DMFD and BDO with the ratio of 1/1.3 (mol/mol) were added into a 1 L round-bottom flask equipped with a condenser, mechanical stirrer, collecting vessel and N_2 inlet. TBT (0.15 mol% of the DMFD) was added to facilitate transesterification. The flask was repeatedly evacuated and purged by highly purified N_2 (99.999%) 3 times. Then the transesterification reaction lasted at 170-200 °C for 4-6 hours, which could be considered to be finished when more than 95% theoretical methanol was obtained. The as-obtained product could be used directly as the hydroxyl terminated oligomer of PBF.

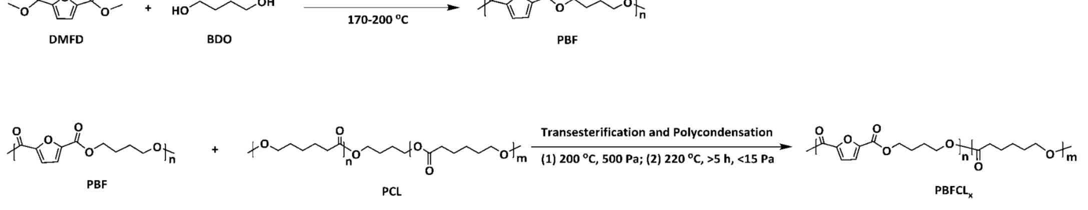

Figure S1. Synthesis routes of PBF and $PBFCL_x$.

Synthesis of polymer $PBFCL_{10}$: Proper amounts of PBF oligomer and PCL diol were added into the reaction container. The targeted copolymer was defined as $PBFCL_{10}$, wherein the subscript 10 meant 10 mol % of CL units in the copolyester. In order to protect the reaction and intermediate products, Sb_2O_3 (0.15 wt%) and antioxidant 1010 (0.1 wt%) were put into the reactor. On the other hand, the pressure of the reaction system was kept at 500 Pa for 200 °C, and then it was further decreased to less than 15 Pa during heating to 220 °C to minimize possible thermal degradation and oligomers sublimation. The progress of polymerization could be evaluated by the torque value of the mechanical stirrer, as the viscosity of the system gradually increased with reaction time. The polycondensation reaction should be lasted for at least 5 h, so that sufficient transesterification between the two oligomers could be ensured. The stable torque value indicated the completion of the polycondensation reaction. Then the product was taken out under the protection of N_2 and cooled down to room temperature. The as-prepared $PBFCL_{10}$ was further purified to remove the residue prepolymers and monomers. The $PBFCL_{10}$ was dissolved in HFP and the solution was stirred at room temperature overnight. Then the solution was dropwise added into the stirring methanol. Such dissolution and precipitation processes were performed 3 times. At last, the $PBFCL_{10}$ was dried in a vacuum oven for 72 h at 50 °C to completely remove the remaining methanol and HFP. The intrinsic viscosity of the as-obtained polymer was recorded with a mixed solvent of phenol and tetrachloroethane (1/1, w/w) in an Ubbelohde viscometer at 25 °C, at the concentration of 0.5 mg/mL.

Section 2. Characterization of the polymer PBFCL$_{10}$

Molecular weights were determined with a Waters 2690 gel permeation chromatography (GPC) using polystyrene standards eluting with tetrahydrofuran. The intrinsic viscosity was recorded by an Ubbelohde viscometer. ^{1}H nuclear magnetic resonance (NMR) spectra were recorded on a Bruker 400 spectrometer at 400 MHz in deuterated chloroform with tetramethylsilane (TMS) as a reference for the chemical shifts. Rheological analysis was conducted on DHR2 (TA Instruments). The basic thermal parameters were measured by DSC (Perkin Elmer Diamond DSC) with a heating/cooling rate of 10 °C/min.

The successful synthesis of PBFCL$_{10}$ with an average molecular weight (M_w) of 67.9 kDa, polydispersity index (PDI) of 1.8, and intrinsic viscosity of 1.1 dL/g was verified by the chromatography and viscosity analysis (**Figure S2**). The high molecular weight and good solubility of the as-synthesized macromolecules in chloroform allow great solution processability for obtaining dense and uniform polymer thin films.

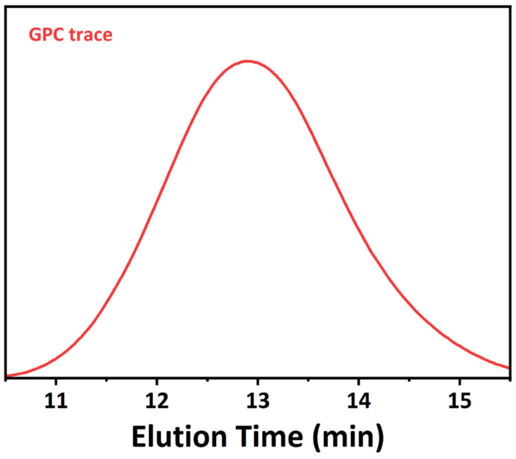

Figure S2. Gel permeation chromatography (GPC) curves of PBFCL$_{10}$

The composition, sequence length and probability of dyads in $PBFCL_{10}$ can be calculated with the ^1H NMR data as shown in **Figure S3** by the following equation,

$$\Phi_F = \frac{I_a}{I_a+I_h} \quad (1)$$

$$\Phi_{CL} = \frac{I_h}{I_a+I_h} \quad (2)$$

$$L_F = \frac{2\Phi_F}{\Phi_{FCL}} = \frac{2I_a}{I_c+I_d} \quad (3)$$

$$L_{CL} = \frac{2\Phi_{CL}}{\Phi_{FCL}} = \frac{2I_h}{I_c+I_d} \quad (4)$$

$$P_{FCL} = \frac{\Phi_{FCL}}{2\Phi_F} = \frac{1}{L_F} \quad (5)$$

$$P_{CLF} = \frac{\Phi_{FCL}}{2\Phi_{CL}} = \frac{1}{L_{CL}} \quad (6)$$

where I_a and I_h belong to the integral intensity of ^1H at the β-position of the furan ring and α-position of the ester group from the PCL monomer, respectively (Equations 1 and 2). Φ_F and Φ_{CL} ascribe to the content of BF and CL unit in $PBFCL_x$, respectively. Φ_{FCL} is the sum of the integral intensity of ^1H at peaks c and d, which are attributed to the -CH_2- group that construct the passage between furan rings. L_F and L_{CL} are the number-average lengths of the BF and CL blocks of the copolymer, and could be obtained from the integral intensity of I_a and I_h through Equations 3 and 4, respectively. P_{FCL} is the probability of a given CL unit with a BF unit on the right, while P_{CLF} is the probability of a given BF unit with a CL unit on the right, both of which can be calculated by Equations 5 and 6, respectively. By virtue of peak assignment in the ^1H NMR spectrum, $PBFCL_{10}$ has an exact composition ($\Phi_{CL,\% mol}$) of 13.7% for the CL segments that is slightly higher than the nominal value but is still experimentally acceptable (Table S1). The number-average lengths (L_F and L_{CL}) of the BF and CL blocks in the copolymers are 7.6 and 1.2, while the probability of finding a CL monomer unit next to a BF monomer unit (P_{CLF} and P_{FCL}) are 0.833 and 0.132, respectively. Such molecular structure

information indicates that each flexible blocks of $PBFCL_{10}$ carry no more than two monomer units of CL, which therefore only acts as gluing agents to connect the adjacent rigid furan-containing BF blocks and make the polymer soluble in the meantime. The small size and minor content of the CL blocks also avoid the occurrence of room-temperature phase separation of the originally incompatible BF and CL components.

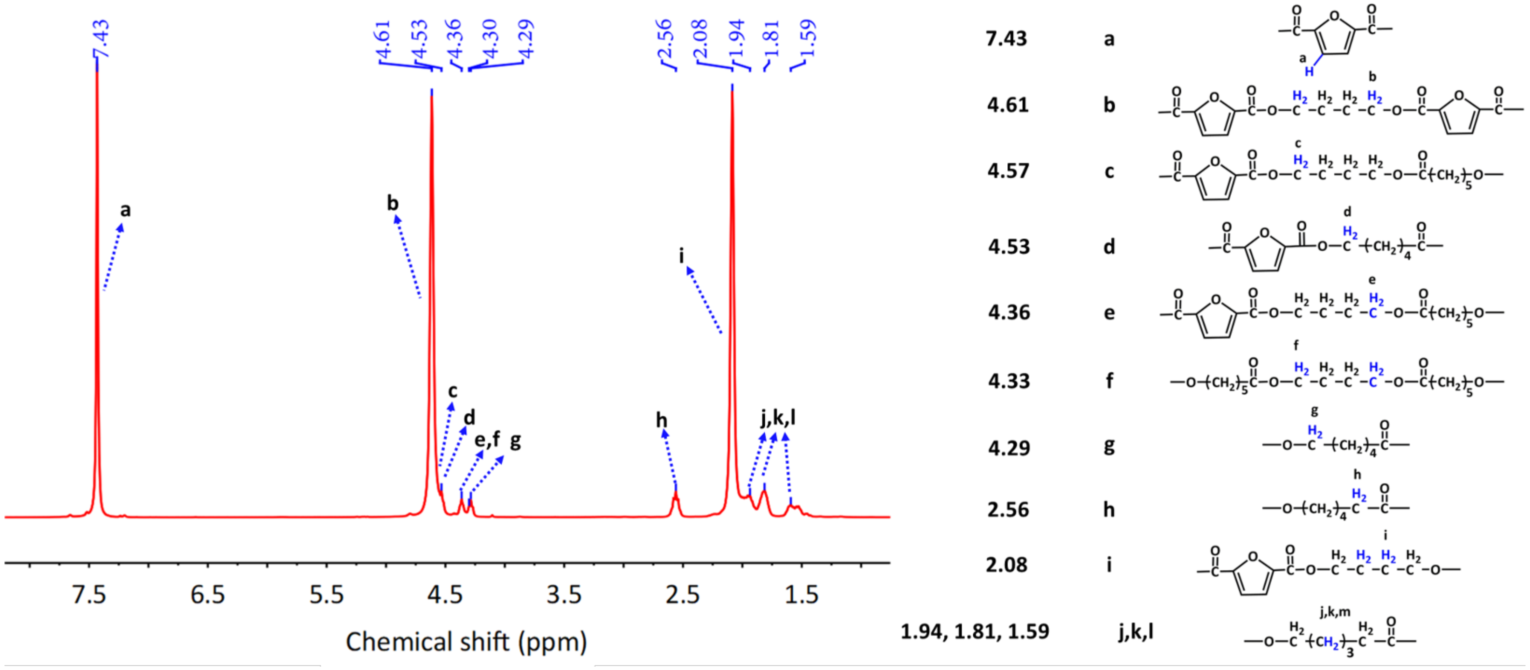

Figure S3. ^{1}H NMR spectrum and peak assignments of $PBFCL_{10}$.

Table S1. The molecular characteristics of $PBFCL_{10}$

Sample	Composition	Sequence Length		Probability of Dyads		Intrinsic Viscosity	GPC	
$PBFCL_{10}$	Φ_{CL} *(mol %)*	L_F	L_{CL}	P_{CLF}	P_{FCL}	[η]	M_w	Đ
	13.7	7.6	1.2	0.833	0.132	1.10	$6.79*10^4$	1.8

As plotted in **Figure S4**, the pseudo constant slope of the frequency-dependent storage modulus and single arc shape of the Cole-Cole curves at low temperatures suggest that micro-phase separation in $PBFCL_{10}$ only occurs when being heated over 180 ºC. Note that the state-of-the-art microelectronic devices and integrated circuits generally work at temperatures not higher than 125 ºC, single-phase material with spatially homogeneous properties will be safely maintained in the $PBFCL_{10}$ thin film and make them thermodynamically stable during memristor device operations.

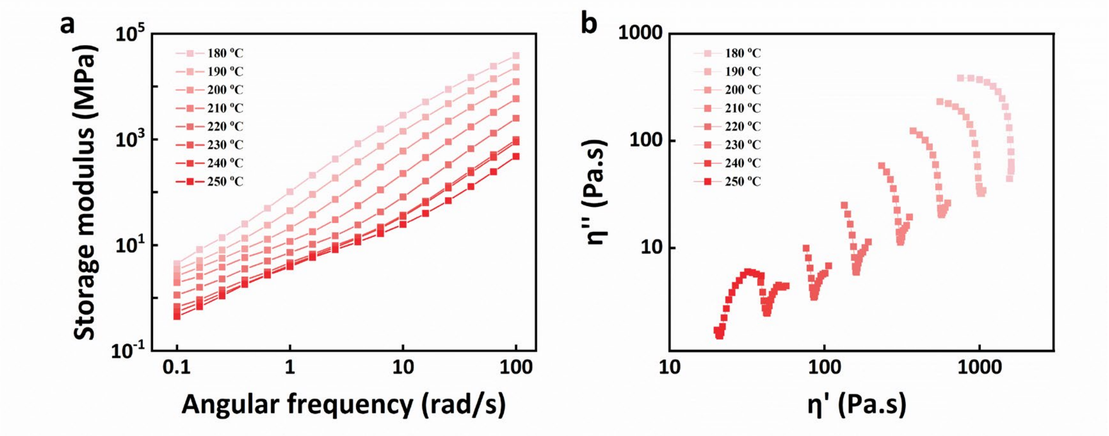

Figure S4. **a** The frequency dependent storage modulus and **b** Cole-Cole curves of $PBFCL_{10}$ recorded at different temperatures.

Calorimetric analysis indicates that the pristine $PBFCL_{10}$ has glass transition, cold crystallization, melting and recrystallization temperatures of 28.4 ºC, 81.1 ºC, 159.5 ºC and 100.4 ºC, respectively (**Figure S5**).

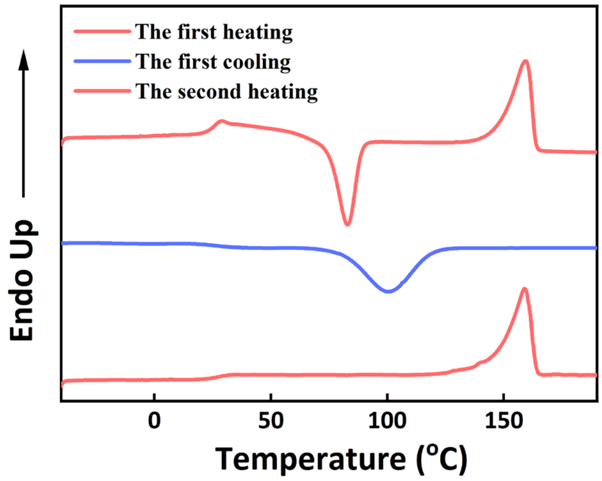

Figure S5. The differential scanning calorimetry (DSC) scan curves of $PBFCL_{10}$ in the first heating, cooling and the second heating procedures, respectively.

Section 3. Biocompatibility of the polymer $PBFCL_{10}$

To realize the emulation of the synapse in biological system, the biocompatibility is a critical index to evaluate the synapse-like electronic device. Cell viability tests were conducted with mouse fibroblast cells (NIH/3T3) to evaluate the biocompatibility of the polymer switching matrix $PBFCL_{10}$. The NIH/3T3 cells were first seeded into a 96-well plate with the density of 5×10^4 cells/ml in 100 μL DMEM (the Dulbecco's modified eagle medium) in each well, and cultured at 37 °C under an atmosphere of 5% CO_2 for 24 h. A 20 μL of $PBFCL_{10}$ solution (2 mg/mL) in chloroform was spin-coated onto the quartz plates. Three pieces of 1×1 cm^2 quartz plates with $PBFCL_{10}$ films (samples 1-3) by different spin-coated rate of 3000, 3500 and 4000 rpm, respectively, as well as a blank quartz substrate (sample 4), were immersed in 1 mL DMEM under the same culturing condition as mentioned above for 24 h to prepare the sample extract solutions. Afterwards, the culture media of four sets of the NIH/3T3 cells in the experimental group were replaced with 100 μL extract solutions collected from samples 1-4, respectively, while the culture medium of the negative control group cells was replaced with 100 μL fresh DMEM. Both the experimental group and control group NIH/3T3 cells were cultured again for 24 h in the incubator at 37 °C under an atmosphere of 5% CO_2. The cellular toxicity of $PBFCL_{10}$ was then evaluated by using a CCK-8 reagent kit. 100 μL of 10% CCK-8 solution was added to each well and incubated for another 3 h. Finally, the absorbance at 450 nm of each well was recorded using a microplate reader (Thermo Multiskan MK3). The cytotoxic experiment was repeated five times and the cell viability was determined by the following equation:

$$\text{Cell viability (\%)} = (A_{sample} - A_{blank}) / (A_{control} - A_{blank}) \times 100\% \quad (7)$$

where A_{sample}, $A_{control}$ and A_{blank} represent the absorbance of the experimental groups that were incubated with the extract solutions of samples 1-4, the absorbance of the negative control group cells that were incubated with fresh cell culture medium DMEM, and the absorbance of the blank CCK-8 containing culture medium, respectively. **Figure S6** reveals that

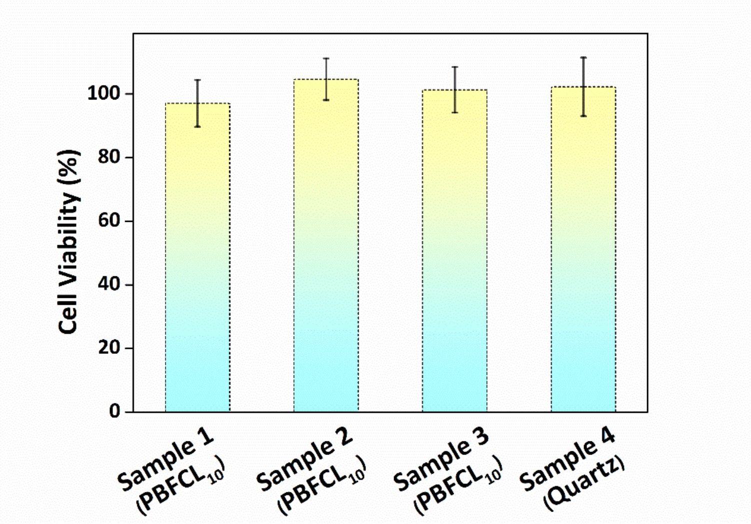

Figure S6. Viability assessment of the NIH/3T3 cells after incubated with extract solutions collected from the $PBFCl_{10}$ thin film-coated quartz substrate samples 1-3 and blank quartz substrate sample 4, respectively. The sample size is 5.

the $PBFCL_{10}$ film has little or no cytotoxicity with the measured cell viability ranging from 97.04 % to 104.61 %.

The physiological states of the cells upon being seeded onto the $PBFCL_{10}$ thin films were further visualized directly with a Leica TCS SP8 confocal laser scanning microscope. Three pieces of 1×1 cm^2 quartz plates with spin-coated $PBFCL_{10}$ films

(samples 1-3), as well as a blank quartz substrate (sample 4), were placed in the wells of a 24-well plate, separately. Then the NIH/3T3 cells with the density of 1 × 10^5 cells/ml in DMEM medium were added into the wells containing samples 1-4, incubated at 37 °C under an atmosphere of 5% CO_2 for 24 h. At the end of the incubation, the culture media were discarded and the samples were washed 3 times with phosphate buffered saline (PBS) solutions. Afterwards, the live and dead NIH/3T3 cells tethered on the sample surfaces were stained with calcein-AM and pyridine iodide, respectively, and incubated for another 30 min. Then samples were washed again with PBS

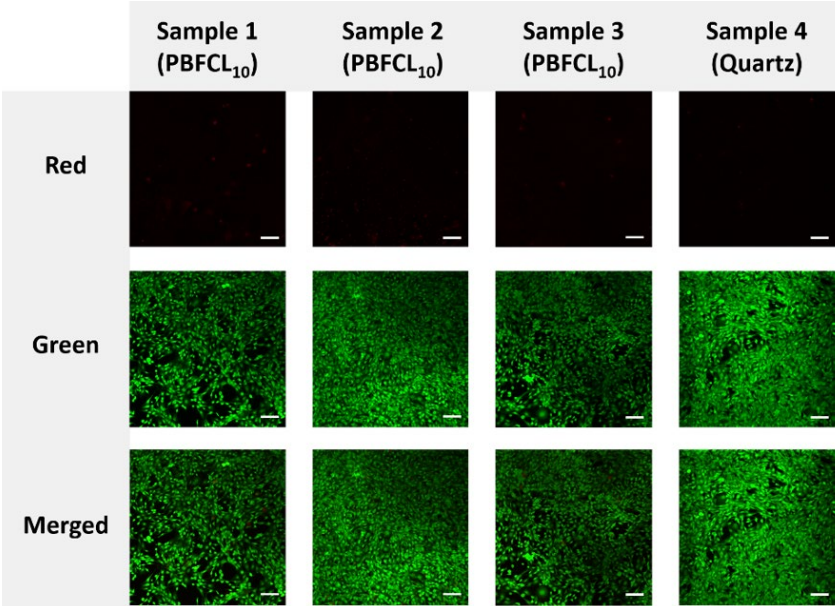

Figure S7. Confocal fluorescence microscopic images of the stained NIH/3T3 cells in red channel for dead cells (upper row), green channel for live cells (middle row) and merged images of the red and green channel (bottom row) for the $PBFCl_{10}$ thin film-coated quartz substrate samples 1-3 and blank quartz substrate sample 4, respectively. Scale bar: 100 μm.

solutions for 3 times to remove the extra dyes. Finally, the proliferation activity and morphology of the NIH/3T3 cells were observed through their stained fluorescence,

green for live and red for dead cells, respectively, with the Leica TCS SP8 microscope. As displayed in **Figure S7**, almost all the NIH/3T3 cells remained live on either the polymer-coated quartz or blank quartz surfaces, suggesting that the $PBFCL_{10}$ polymer does not exhibit obvious cytotoxicity and is thus biocompatible.

Section 4. Morphological property of the $PBFCL_{10}$ film

During the device fabricating process of Au/$PBFCL_{10}$/Ag, the spin-coated nanofilm displays a smooth characteristic with 0.39 nm root-mean-square (RMS) roughness from the atomic force microscope (AFM) characterization as shown in **Figure S8**. This characteristic with physically even $PBFCL_{10}$ film is the fundamental principle for successfully fabricating the 32 × 32 Au/$PBFCL_{10}$/Ag crossbar array as mentioned in METHOD.

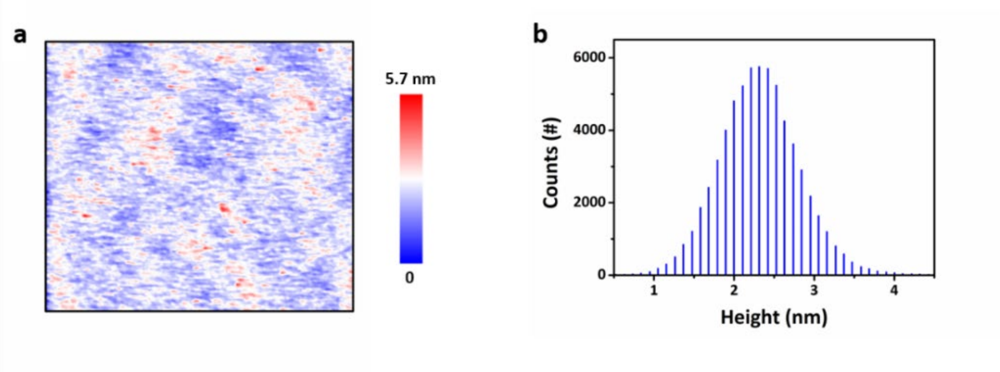

Figure S8. a The AFM measurement for the $PBFCL_{10}$ film surface characterization. **b** The height distribution of the $PBFCL_{10}$ film from Figure S8a.

Section 5. Zoom-in mode of the Au/PBDCL10/Ag device

The zoomed-in mode by scanning electron microscope (SEM) clearly displayed the local crossbar device structure, which resembles the synapse structure with presynaptic membrane, synaptic cleft, and postsynaptic membrane (**Figure S9a**). Furthermore, the transverse structure of Au/$PBFCL_{10}$/Ag displays that the device's nanofilm thickness has reached 44 nm (**Figure S9b**).

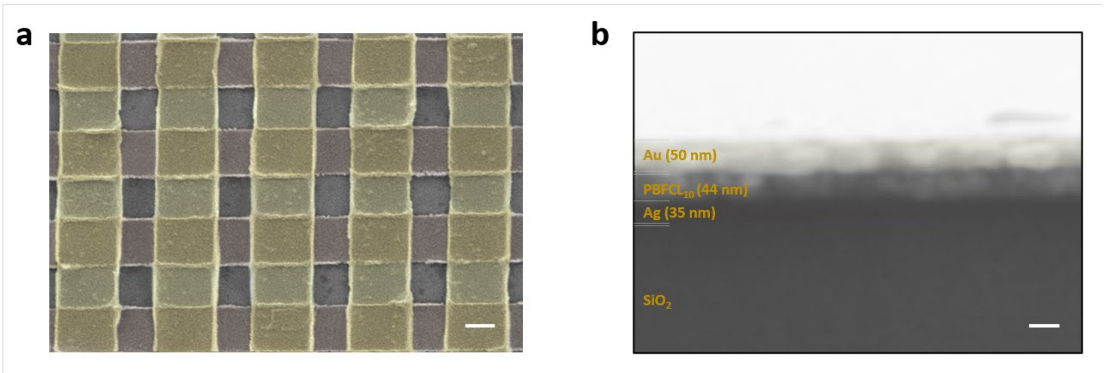

Figure S9. a The zoom-in mode (Bar scale: 1 μm) and **b** cross-section structure (Bar scale: 50 nm) of Au/$PBFCL_{10}$/Ag memristive device.

Section 6. Modulation of self-protective depression plasticity

As shown in **Figure 2b** and **2c**, the conductance increases with successive 30 pulses (amplitude: 2.3 V, width: 10 μs and interval: 40 μs) will cause the LTP effect with conductance rising from 240.6 to 1251.4 μA, while continuously stimulating the device by pulse, an unusual phenomenon of conductance decreases from 1251.4 to 232.2 μA can be observed with a self-protection depression (SPD). An LTP relationship has fitted as below with an exponential function (R^2=0.999) (**Figure S10**).

$$y = A \times \exp(-x / t) + y_0$$

where A is a constant of 1580.0, x is the applied pulse counts, t is the time constant of the LTP curves and derived as 23.95 and y_0 is the bias value of the function.

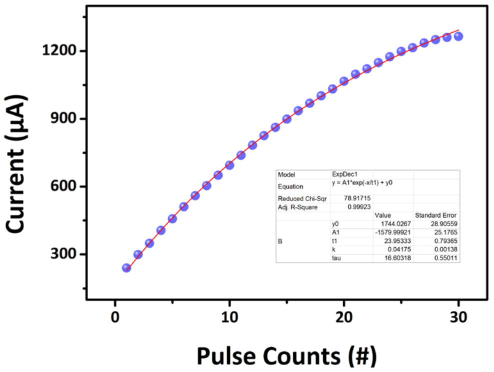

Figure S10. The fitted function during the process of LTP effect.

Interestingly, the decreased extent of the self-protective characteristic can be modulated by regulating the pulse interval. Compared with the pulse interval of 40 μs with 29 pulses, higher frequency pulses with 20 and 30 μs will result in a faster conductance decrease with 20 and 24 count pulses back to the low conductive state,

respectively (**Figure S11**). Reversely, a lower frequency pulse with 80, 120, and 200 μs interval can induce a slower conductance decrease amplitude (**Figure S12**). The conductance variation can be fitted by a linear function containing (y = 1272.93 - 48.72 x), (y = 1281.90 - 41.03 x), (y = 1294.69 - 35.15 x), (y = 1285.58 - 20.62 x), (y = 1232.93 - 9.29 x) and (y=1248.78 - 0.52 x) for 20, 30, 40, 80, 120 and 200 μs time interval, respectively. The summary current variation result by the different pulse interval has shown in **Figure S14**.

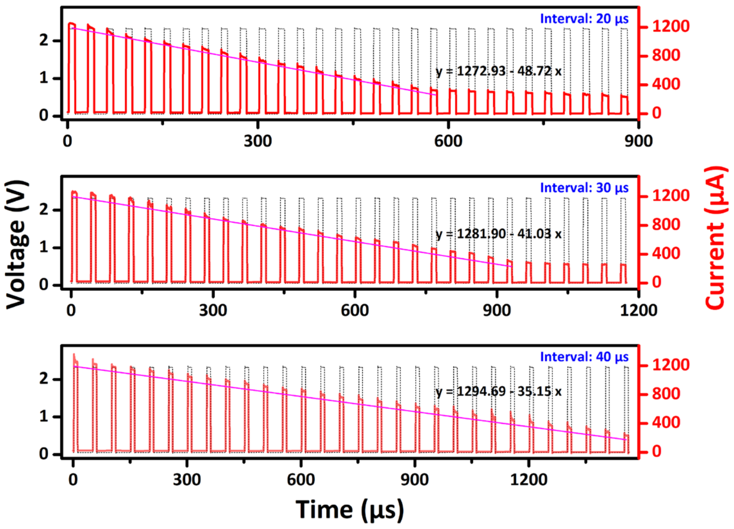

Figure S11. The current variation amplitude with the pulse interval (top: 20 μs, middle: 30 μs, and bottom: 40 μs) during the process of SPD effect.

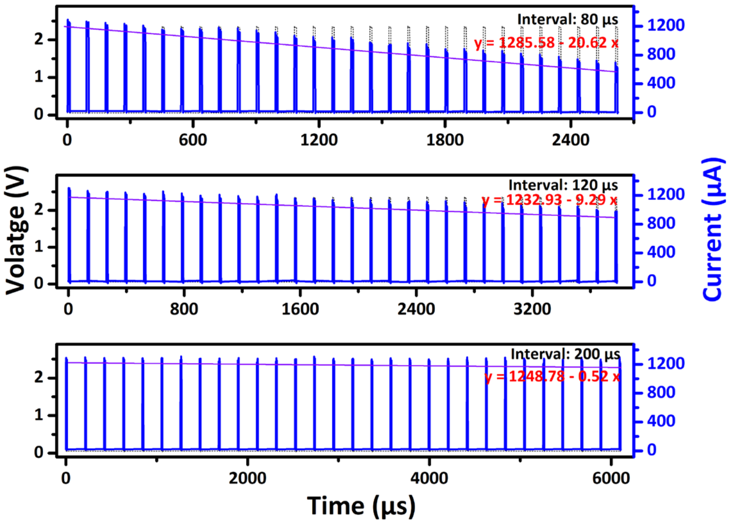

Figure S12. The current variation amplitude with the pulse interval (top: 80 µs, middle: 120 µs, and bottom: 200 µs) during the process of SPD effect.

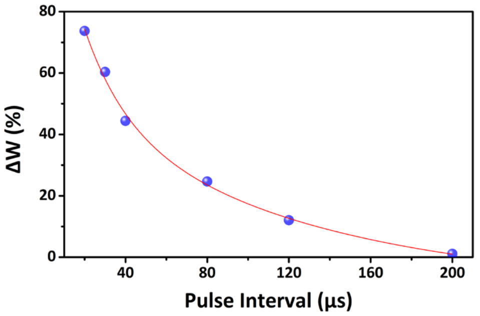

Figure S13. The SRDP emulation during the SPD modulation process.

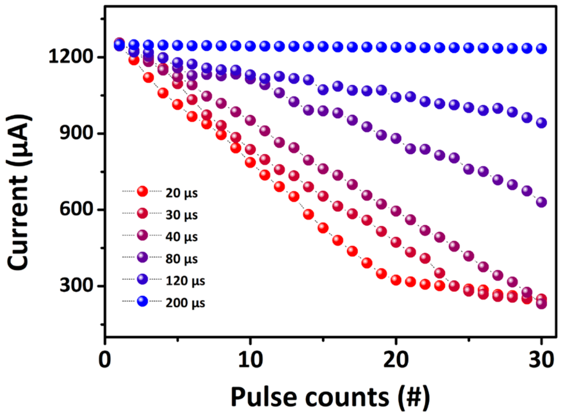

Figure S14. The peak current variation modulated by different pulse interval (20, 30, 40, 80, 120, and 200 µs).

Section 7. The simulation results for the in-memory two-factor authentication (IM-2FA) achievement.

With utilizing the characteristic of the self-oscillated synapse to tune the weight after the network's training (**Figure 2e**), the IM-2FA can be realized. The structure of the ConvNet used to achieve IM-2FA is shown in **Figure S15**. The network includes two convolutional layers, two pooling layers, and two fully connected layers used for handwritten digit recognition.

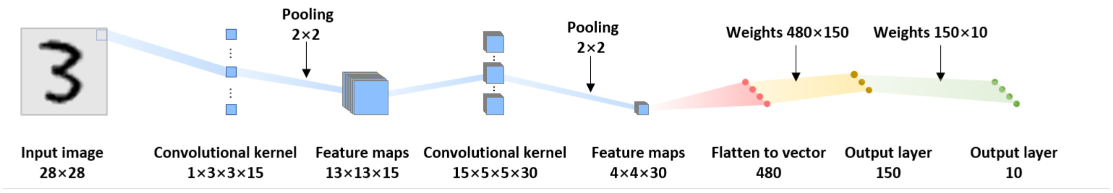

Figure S15. The schematic diagram of ConvNet structure.

The weights mapping (1.00E-05 to 1) of the ConvNet to the conductance (239.9 to 1264.4 μS) is shown in **Table S2**. Each weight of the ConvNet is modulated according to the plasticity of the self-oscillated synapse, in which each cell in the matrix will be gradually tuned. To scrutinize the potential of the ConvNet for the target recognition task, the confusion matrix has shown in **Figure 5d** with the accuracy as well as loss function evolution during the network's training process (**Figure S16**). It should be noted that the condition of the zero epoch represents the network's performance at the pristine time with the random weight generation.

Table S2. Weight-conductance mapping rules

No.	Synaptic Weight	Device Conductance (μS)
1	1.00E-05	239.9
2	0.034	299.3
3	0.069	349.2
4	0.103	406.2
5	0.138	457.7
6	0.172	511.1
7	0.207	559.7
8	0.241	604.3
9	0.276	650.1
10	0.310	694.3
11	0.345	738.7
12	0.379	783.1
13	0.414	825.5
14	0.448	862.4
15	0.483	898.8
16	0.517	935.8
17	0.552	968.7
18	0.586	1002.0
19	0.621	1032.0
20	0.655	1066.3
21	0.690	1097.6
22	0.724	1121.2
23	0.759	1148.9
24	0.793	1174.9
25	0.828	1198.3
26	0.862	1214.7
27	0.897	1235.8
28	0.931	1250.4
29	0.966	1260.1
30	1	1264.4

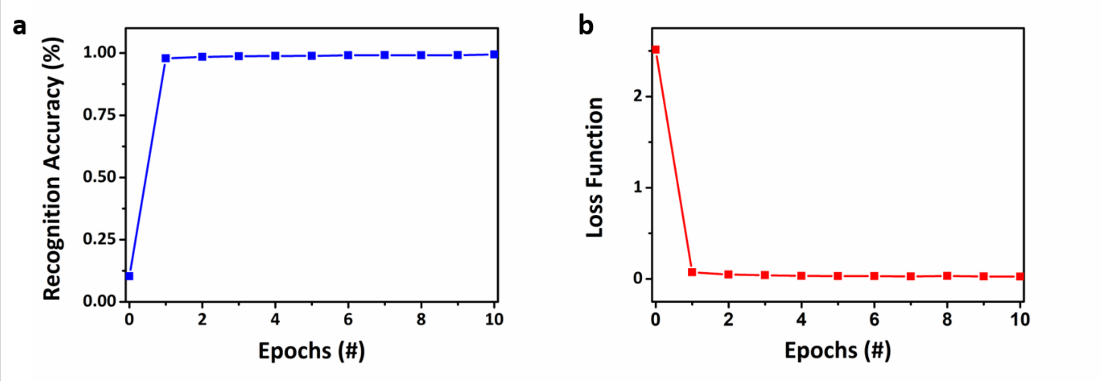

Figure S16. The evolution of **a** recognition accuracy and **b** loss function across the epochs of the network.
